# Applying Structural Systems Thinking to Frame Perspectives on Social Work Innovation

**DOI:** 10.1177/1049731516660850

**Published:** 2016-08-06

**Authors:** Erin J. Stringfellow

**Affiliations:** 1 Brown School of Social Work, Washington University in St. Louis, St. Louis, MO, USA

**Keywords:** social work research and training, innovation, structural systems thinking, system dynamics

## Abstract

**Objective:**

Innovation will be key to the success of the Grand Challenges Initiative in social work. A structural systems framework based in system dynamics could be useful for considering how to advance innovation.

**Method:**

Diagrams using system dynamics conventions were developed to link common themes across concept papers written by social work faculty members and graduate students (*N* = 19).

**Results:**

Transdisciplinary teams and ethical partnerships with communities and practitioners will be needed to responsibly develop high-quality innovative solutions. A useful next step would be to clarify to what extent factors that could “make or break” these partnerships arise from within versus outside of the field of social work and how this has changed over time.

**Conclusions:**

Advancing innovation in social work will mean making decisions in a complex, ever-changing system. Principles and tools from methods that account for complexity, such as system dynamics, can help improve this decision-making process.

In January 2016, the field of social work officially launched its Grand Challenges Initiative (Society for Social Work and Research, 2016), the culmination of nearly 4 years of planning beginning in 2012 (Uehara et al., 2014, 2013). The goal is to galvanize researchers, practitioners, and educators to focus their efforts on a range of seemingly intractable problems from social isolation to homelessness to income inequality. This initiative is set within the broader context of an ongoing discussion about social work and science (Brekke, 2012; Gehlert, 2016) and the role of innovation in addressing these challenges.

The purpose of the Grand Challenges Initiative has been framed as an agenda for developing social innovations (Sherraden et al., 2015). Among the more than 80 challenges originally suggested, the 12 eventually chosen by the executive committee require, among other criteria, innovations in science, technology, and practice (Uehara et al., 2014, 2013). Indeed, innovation is clearly central to the success of the Grand Challenges Initiative.

The Grand Challenges Initiative and the ongoing discussion about science and social work provided a backdrop for the 2015 Innovation in Social Work Roundtable, an opportunity for 19 social work doctoral students and junior and senior faculty researchers to write about and discuss innovation in social work. Prior to the roundtable, attendees were tasked with writing concept papers related to one of the six topics. At the 2-day round-table, participants engaged in structured discussion regarding these topics, which served as the basis for the other articles published in this issue. As the roundtable progressed, it became apparent that several recurring themes arising in the concept papers warranted further discussion. Based on these conversations, this article uses heuristic tools based in system dynamics (Forrester, 1961; Sterman, 2000) to identify linkages across the concept papers and suggest next steps based on these initial insights, including consideration of how system dynamics principles can guide the future of innovation in social work.

Innovation does not necessarily, and indeed maybe rarely, involve the development of completely new tools or methods; more often, innovation is the application of existing ideas in new contexts. This article is an example of using existing ideas in a new context, and thus to guide comprehension of the analysis some key terms need to be defined.

## Complex Adaptive Systems

Complex adaptive systems are systems in which the interrelated components of the system adapt over time in response to changes in that same system (Miller & Page, 2007). In a complex adaptive system, such as the field of social work, the interrelated components are researchers, practitioners, and educators, or depending on the perspective taken, the institutions with which they are associated. Regardless of where the boundaries drawn, the entities in the system adapt based on their perception of the current state of affairs in that system.

Interdependencies are introduced when actors in a system act based on the perceived state of that system. This is what makes a complex system different from a complicated system. When a system is complex, removing or changing individual components changes the functioning or behavior of the system. Thus, it matters what researchers do, because that results in a different system, which then changes the behavior of educators and practitioners, which also changes the system and therefore researchers' actions. In a complicated system, on the other hand, the components are independent of one another and thus removing one component does not change the behavior of the system (Miller & Page, 2007). These interdependencies make it very difficult if not impossible to anticipate the outcomes of any one component's decisions on the overall system. Making decisions as if these interdependencies did not exist can contribute to or even recreate the very problems that are supposed to be addressed (Sterman, 2006). At the least, then, decisions should be made with awareness of these interdependencies.

## The Structure–Behavior Relationship

System dynamics, which originated in engineering, is one tool that decision makers can use to account for interdependencies in complex adaptive systems (Forrester, 1961). System dynamics is increasingly used in health and social sciences, including social work (Hovmand, 2014; Hovmand, Ford, Flom, & Kyriakakis, 2009; Munar, Hovmand, Fleming, & Darmstadt, 2015), public health (Fallah-Fini, Rahmandad, Huang, Bures, & Glass, 2014; Hirsch, Homer, Trogdon, Wile, & Orenstein, 2014; Homer & Hirsch, 2006), and psychology (Wittenborn, Rahmandad, Rick, & Hosseinichimeh, 2016).

This article relies on structural systems thinking as its framework. The term *structural* is used because system dynamics is based on the principle that all dynamic, that is, adaptive, systems share a common structure and that structure determines behavior (Forrester, 1961, 1968b). Structure is an important addition to systems thinking, which too often is interpreted as everything is connected to everything, a truism of limited use when the goal is to solve problems. Another way to think of structure is in terms of an iceberg: Events are at the top, but analysts need to move below the surface to identify patterns. Deeper still is the structure driving those patterns; finally, at the base, are the values influencing the structure.

There are three structural elements that drive the state of a system: activity, the accumulation of that activity over time, and feedback loops that either attenuate or amplify activity in the system (Forrester, 1968a, 1968b; Richardson, 2011). Accumulations can rise and fall due to the rate of activity. Feedback effects contribute to delays in or resistance to change, unintended consequences, and out-of-control growth and decline (Richardson, 1999, 2011). Feedback effects, good or bad, arise from within the system rather than due to external forces (Sterman, 2000). The state of the system, then, is a snapshot of the accumulation of activity at any given moment.

## Two Ways of Showing Structure

**Figure 1:**
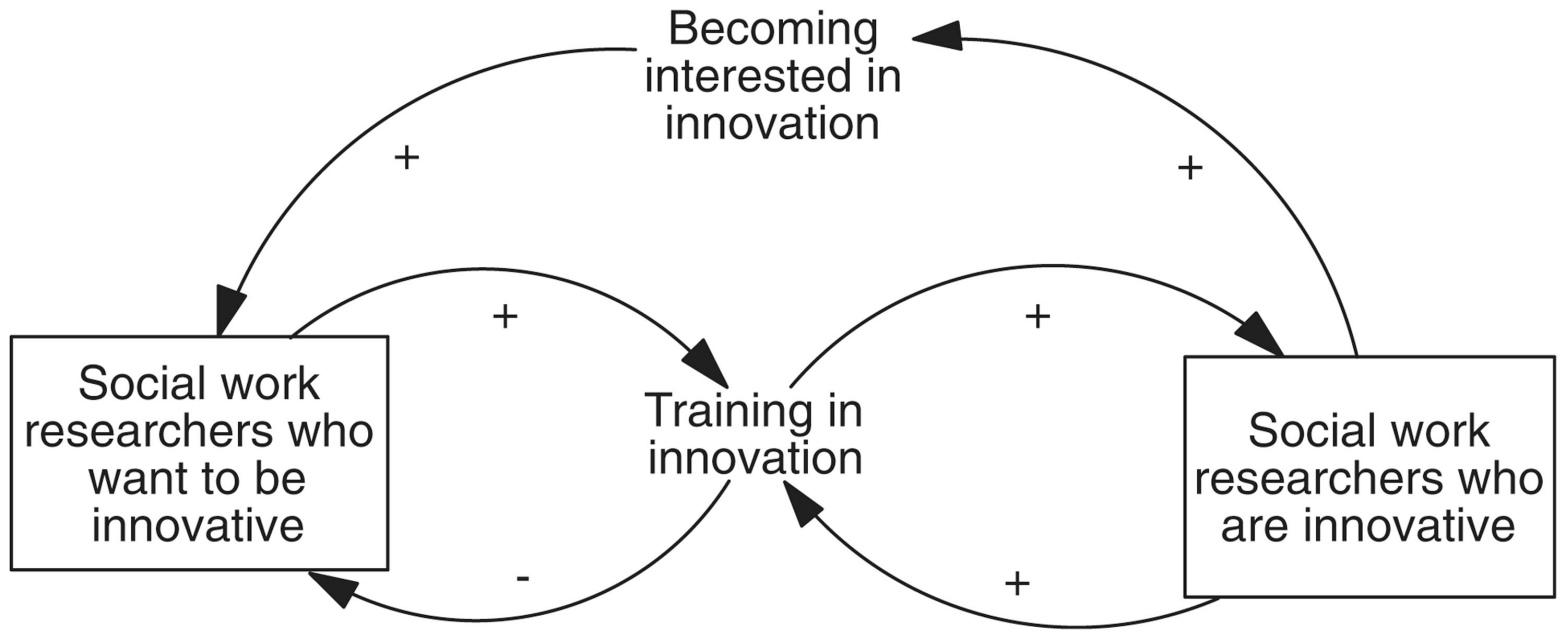
Developing innovative researchers causal loop diagram.

In system dynamics, there are two diagramming techniques for depicting the structure of a system. The first is causal loop diagrams (CLDs), which make the feedback nature of systems explicit through the use of loops. Figure 1 is a CLD depicting a simplified way of thinking about how social work researchers become innovative. It is simplified because it places social work researchers into two groups—those who want to be innovative and those who are innovative—whereas the level of innovation among researchers in reality falls along a continuum. For this example, consider how activity results in accumulations, which are shown in boxes. An important point to remember is that the terminology and diagramming conventions used in system dynamics are just a way to describe and depict what is actually happening in all dynamic systems.

Figure 1 is interpreted as follows. As interest in innovation increases, which is an activity, there are more social work researchers who want to be innovative, which is an accumulation; the relationship is indicated by a positive (+) sign. Social work researchers who want to be innovative are affecting the system by increasing the activity of training in innovation, which over time increases the number of social work researchers who are innovative. However, a less intuitive effect of training in innovation is because it flows from one group to the other, from wanting to be innovative to being innovative, as the rate of training increases it pulls from the group of social workers who want to be innovative; that relationship is therefore represented by a negative (−) sign because the effect is in the opposite direction.

Thus, at any given moment, the total number of social work researchers who want to be innovative is represented by an accumulation; mathematically, it is the difference between the rate at which researchers' interest is increasing and the rate at which researchers are being trained plus the number of individuals who were interested but have not yet been trained. This has implications for the state of the system. For instance, it is likely that the current rate at which social work researchers are becoming interested in innovation (an activity) is increasing, whereas the rate at which they are being trained is probably much slower. Thus, the total number of social work researchers who want to be innovative will continue to rise until training outpaces increasing interest. This is why system dynamicists posit that activity accumulates over time.

There are other effects that social work researchers who are innovative can have on the system. For instance, they can increase their ranks by virtue of the fact that they can train researchers who want to be innovative. Another effect they can have is by increasing interest in innovation. These two effects are positive feedback loops.

**Figure 2:**
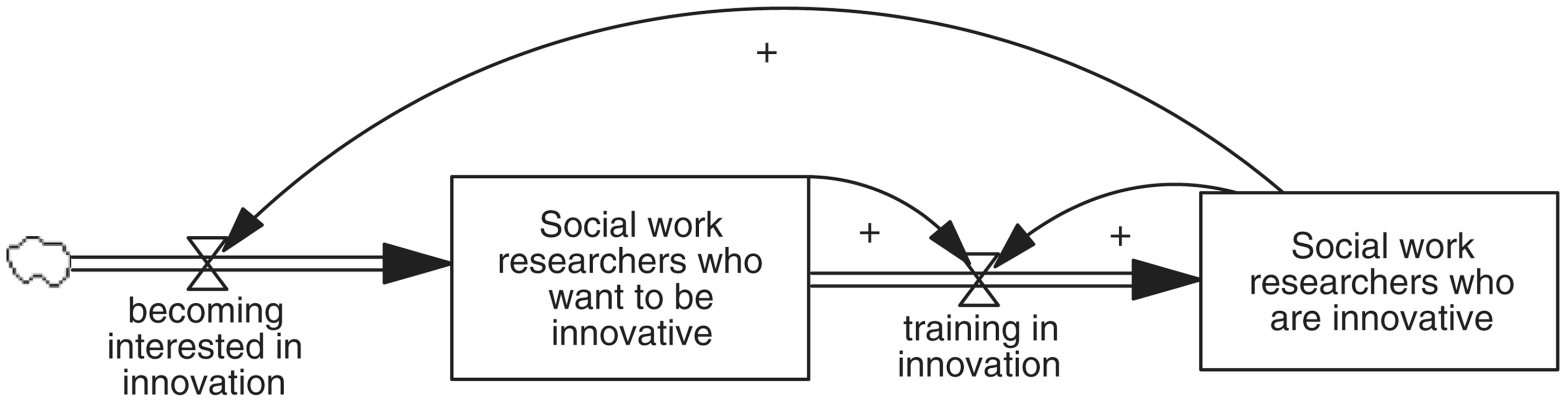
Developing innovative researchers stock-and-flow diagram.

The second way to depict these structural relationships is through a stock-and-flow diagram (SFD; Figure 2). Compared to CLDs, SFDs more precisely differentiate between activities (flows), the accumulation of those activities (stocks), and the effects of those accumulations on the system (feedback loops). For instance, Figure 2 makes it clear that researchers flow from one group or stock to the next; the SFD version also makes it clearer that, whereas social work researchers who want to be innovative represent the difference between two activities, training and becoming interested, social work researchers who are innovative is simply the accumulation of training. Their numbers are not decreased by training others or influencing others to become interested in innovation; in this diagram, there is no outflow from their group.

However, CLDs and their emphasis on feedback loops rather than activity and accumulations are more intuitive to those unfamiliar with system dynamics, so they are used in this article. Nonetheless, it is important to keep in mind the difference between activity and the accumulation of that activity over time.

## Mental Models

The final term that needs to be introduced is *mental models*, which are people's rich and complex ideas about why problems are occurring in dynamic systems (Doyle & Ford, 1998). Mental models reflect the limited knowledge available to people (i.e., bounded rationality) and assumptions about how parts of the system interact with one another; they do not necessarily represent reality, facts, or the truth. Nonetheless, mental models are important because they form the basis on which decisions are made. More importantly, mental models can always be improved; indeed, that is a key goal of system dynamics modeling (Sterman, 2000).

Eliciting mental models through qualitative modeling is one way to improve mental models (Coyle, 2000). There are heuristic tools used in system dynamics to accomplish this goal, including clarifying how the problem is defined in terms of change over time, considering what causal and feedback effects could be creating the problem or could help solve it, and constructing CLDs and SFDs to reflect these mental models (Ford & Sterman, 1998; Hovmand, 2014). These steps increase insight by encouraging people to be explicit about what they think is contributing to or could help solve the problem of interest. The most insight is typically gained through simulation, which shows participants the effects of multiple intersecting components in the system, which are often counterintuitive (Homer & Oliva, 2001).

This article uses concept papers from 19 social work faculty members and doctoral students to demonstrate how using these heuristic tools qualitatively can provide insight and prompt important questions regarding innovation in social work. Thus, the results are written not as a statement of fact but rather as the author's best interpretation of the collective mental model of participants. Finally, suggested next steps are offered based on the heuristic tools and principles of system dynamics.

## Method

### Data

The data used to construct the CLDs are from the 19 concept papers written by the Innovation in Social Work Roundtable participants prior to the roundtable. Each participating faculty member (*n* = 6) wrote a two-page concept paper summarizing his or her views on a specific topic related to innovation in social work. The topics were research–practice partnerships, responsible innovation and social work science, training for scientific careers, innovations in the profession of social work practice, community-based and interdisciplinary research, and the clash or convergence between innovation and social work. Two or three (primarily) doctoral students (*n* = 13) were assigned as discussants to each concept paper; their responsibility was to respond, also in writing, to these concept papers. All of the concept papers were made available to participants prior to the roundtable.

### Analysis

Most of the concept papers fit the definition of purposive text data, which arises when expert stakeholders with sophisticated knowledge of a system have a “focused discussion on the problem or system at hand” in a frank way that reflects their mental models of the system (Kim & Andersen, 2012, p. 312). The mental models of some participants came through more clearly than those of others, primarily due to what they used as their primary information source. Personal experience and observation are richer sources of information for eliciting mental models than extant literature, unless participants make clear how they interpret extant research and to what extent it informs their own decision-making.

The text of the concept papers was coded to elicit causal structures as articulated by the participants using a modified approach described in system dynamics journals by Kim and Andersen (2012) and Turner, Kim, and Andersen (2013). The overall approach was to identify causal linkages within and across papers and then merge variables and causal linkages to create CLDs. A coding chart was created in Excel for each concept paper, which included the cause-and-effect variables posited by the participant as important for understanding the particular problem related to innovation. After each concept paper was associated with a coding chart, they were grouped into their original six sets (one faculty member and two or three students) to create six CLDs in Vensim PLE (Macintosh Version 6.3), a system dynamics software program, that linked and combined concepts. Because student papers were written in response to faculty papers, it was typically clear how the same concepts were being used, built on, or modified in a given set of papers. Color coding and other markers were used to distinguish between concepts and links derived from one versus more than one paper.

This process was completed for each set of concept papers, resulting in six CLDs whose initial versions had up to 68 variables. CLDs are iterative products and thus each version had fewer or combined variables based on similar concepts. Concepts and links were merged across the six CLDs to create one comprehensive but unwieldy CLD. The CLD was made more digestible by identifying themes in the model, which form the basis of the results. However, presenting each CLD theme was not an option. Thus, the final step was to reconstruct the model tied directly to the text, which is shown in the results. However, readers are encouraged to sketch their own CLDs while reviewing the results.

## Results

Table 1 summarizes the presence of the overarching themes across the six CLDs that reflected the 19 concept papers. Considering the breadth and depth of the material covered by participants, it is likely that the authors missed some coverage of these themes. Two themes are not shown in Table 1 but arose frequently. First, participants noted the goal of innovation should be to improve community well-being or health, or put another way, that improving communities' health and well-being is the mission of social work and should therefore be the goal of innovation. The term *community health and well-being* is a catchall for many concepts used by participants, including community capacity to respond to challenges, meeting needs, societal impact, and common good. Second, several participants referenced how social work could uniquely contribute to the success of innovative research, focusing on its ethos, holistic view, philosophical tradition, and ability to address questions of politics and power.

Most often, innovation referred to learning new methods, expanding theoretical knowledge and utilization, and developing new solutions to problems. Innovation in relation to teaching typically referred to the extent to which innovations derived from research and practice were transferred to the classroom or field practicum.

As a reminder, the language used in this discussion reflects the mental models of the participants, or what participants stated were important factors in innovation, and thus results should be interpreted as such.

### Outside Factors Changing Academic Expectations to Be Innovative

A reframed research agenda focusing on integrated, multilevel research is pushing academia toward innovation and new collaborations. The sources of this reframed agenda are threefold. First, the ratio of public to private funding is decreasing with constraints in National Institutes of Health funding and a concomitant shift to private funding; second, there is an increasing gulf between current community health and well-being and expectations for health and well-being, which is increasing the urgency to find innovative solutions; and third, the gulf in community health and well-being is increasing recognition of the need for local, context-specific, culturally relevant solutions, which often require innovation.

### Partnerships and Collaborations as a Key Mechanism for Innovation

Participants had clear ideas about what is needed to improve community health and well-being beyond being innovative. These ideas focused on partnerships and collaborations.

The responsibility to solve complex, wicked problems does not fall on social work alone. Hence, the reframed research agenda will move forward regardless of social work's role. This requires much more expertise than can be found within a single discipline, let alone a single researcher. Thus, multiple types of partnerships and collaborations will need to develop, including transdisciplinary partnerships; partnerships with communities, clients, and practitioners; and although less discussed, collaborations that include academia and social innovators such as venture capitalists and benefit corporations. The more partnerships there are, the greater the number of successful partnerships, all else being equal. Thus, social work researchers cannot achieve innovation without working with others; however, multiple factors could “make or break” the success of these partnerships.

### Success of Transdisciplinary Partnerships

Participants stated that more transdisciplinary partnerships increase the number of successful research teams, all else being equal. Clearly, however, all else is not equal. Specifically, these partnerships succeed as a function of members' transdisciplinary-specific competencies (National Research Council, 2014). Transdisciplinary competencies include deep functional knowledge and expertise, a broad understanding of substantive areas outside personal expertise, strong communication skills, creativity, and empathy. The ability to manage power imbalances also increases the success of these partnerships and is an area in which social work researchers have the potential to contribute their unique skills.

### Success of Partnerships With Practitioners and Communities

Communities include constituencies, neighborhoods, families, and individuals with whom practitioners work or for whom they advocate, develop policies, target for interventions, and so on. Practitioners include social workers and other professionals with whom communities and researchers work. Communities and practitioners are discussed together for simplicity; however, although both have less power relative to researchers, practitioners typically have more power than communities and are subject to different power imbalances.

Growing recognition of the need for local, context-specific, culturally relevant solutions has an unclear effect on researchers. At the least, it could lead more researchers to attempt to develop partnerships with communities and practitioners. The extent to which researchers are able to form any partnerships likely depends on community and practitioner willingness to participate in research, which may be low due to memory of previous exploitation, impatience with research's slow pace, and perceived lack of relevance of research.

If partnerships develop, success in developing ethical partnerships is contingent on researchers being able to manage power imbalances and practice reflexivity. Ethical partnerships necessitate addressing the community's needs and acknowledging historical exploitation by researchers. Only with the very slow passage of time does the memory of this exploitation begin to decline. If power imbalances are not addressed, communities' memory of exploitation increases and participation in research remains low.

If ethical partnerships are developed with communities and practitioners, participants believe these collaborations will have several important benefits. First, working directly with communities and practitioners improves theory and knowledge, and thus the quality of innovations. Second, ethical partnerships result in research that has greater relevance for communities and practitioners, which increases the use of innovations resulting from these partnerships and increases their willingness to participate in future research. Finally, the combination of greater use of innovations that are of higher quality is believed to have a positive impact on community health and well-being.

Although most of these discussions were from the perspective of social work researchers, the principles apply to any researchers engaging in researcher–community partnerships. Thus, as with transdisciplinary partnerships, social work researchers should expect that community and practitioner partnerships will grow regardless of social work's role.

### Factors Relevant to Social Work Master of Social Work (MSW) and PhD Training

Thus far, the discussion has described the factors driving innovation, highlighted how partnerships will be essential to accomplish this, and discussed the make-or-break factors in different partnerships that could affect their success. Managing power imbalances on research teams and with practitioners and communities is one area to which social work researchers can and should contribute; however, social workers also need new skills.

In social work, recognition of the larger push toward innovation is increasing encouragement of students and faculty members to broaden, collaborate, experiment, and integrate, that is, innovate. Through various mechanisms including exposure to transdisciplinary teams, this encouragement has the potential to increase the number of social work researchers who have transdisciplinary competencies, such as substantive knowledge outside their personal area, creativity, and communication skills. However, pressures to specialize and deepen at the MSW and PhD level push against this encouragement to innovate. To a certain extent, specialization is needed to gain solid training in one's home discipline, another transdisciplinary competency, and to avoid practitioners developing low-quality innovations uninformed by substantive expertise. However, developing transdisciplinary competencies and other intellectual habits of mind could require a level of time and support in PhD programs not afforded to many students.

Yet developing these competencies could increase social workers' confidence on transdisciplinary teams, an important factor raised by several participants. There is concern that pressures to specialize contribute to within-discipline fragmentation and training that is not meeting the expectations of MSW students. This dampens confidence and willingness to take risks, which makes it harder for social work to draw on its unique contributions, lead by example in developing transdisciplinary teams, and become the academic home of social innovation. Being the academic home would involve collaborations within and outside of academia, with the goal of developing higher quality innovations that are currently being created by innovators, who are largely detached from the scientific enterprise of knowledge development.

### CLD With All Major Themes

**Figure 3:**
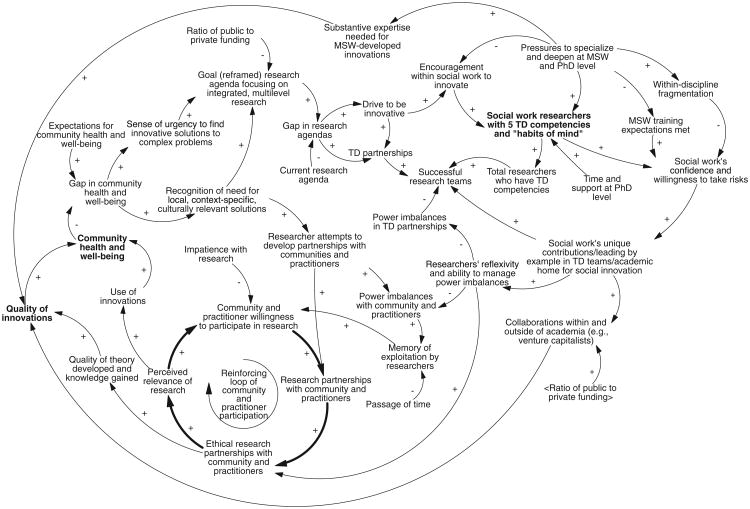
Causal loop diagram (CLD) summarizing major themes from concept papers. This CLD has many “open loops” that would need to be closed in order to identify feedback loops. This ideally would occur through ongoing dialogue.

Figure 3 is a CLD capturing the aforementioned themes. For visual clarity, boxes were not drawn around variables to distinguish activity from accumulation as shown in Figure 1. However, while examining the CLD, readers are encouraged to consider where such distinctions are important. Three variables are rendered in bold font to draw attention to points of future discussion: social work researchers with five transdisciplinary competencies and habits of mind, quality of innovations, and community health and well-being.

CLDs are almost always overwhelming at first. To read this diagram, identify a variable and follow it around the loop until it comes back to the original variable or reaches a dead end. For instance, the variable of community and practitioner willingness to participate in research increases researcher partnerships with community and practitioners, which increases ethical partnerships, which increases the perceived relevance of research, and which increases community willingness to participate in research—hence closing the loop. This is a reinforcing feedback loop, because the original variable reinforces itself as it feeds around the loop. It can go in the opposite direction as well; as community willingness to participate declines, partnerships decline, and so on.

As currently conceptualized, many loops are open-loop dead ends; they do not feedback around to themselves. However, readers may be readily able to perceive potential additional links that would close these loops. Because this is easy to do but would go beyond the content of the concept papers, links not described in the original papers were not added.

## Discussion

System dynamics emphasizes how activity, the accumulation of that activity over time, and feedback loops amplifying or attenuating that activity produce the current state of a system. Understanding how this activity interacts across components of a system is a key step to making better decisions. However, this requires clarifying the dynamic problem of focus, the boundaries of the system, and identifying limits to growth—a certain type of feedback—and not just feedback that leads to growth. These are the key questions for social work moving forward as it considers innovation and are worth consideration by individuals, groups, and institutions.

### Clarify the Dynamic Problem (and Thereby the Goal)

Concept papers centered on whether current solutions are accomplishing what they should with regard to community health and well-being, the challenge of developing innovations themselves, and defining social work's role in creating innovative solutions. Thinking more explicitly about which of these is the core problem of interest would focus future discussion on the most relevant issues. Thus, a suggested next step would be to graphically depict the key problem as it has changed over time, going back as far as is necessary, for example, 100 years, to understand its dynamic nature. Plot key inflection points and exponential rises and declines and think about key historical changes. This will also help define the goal more clearly.

The concept papers seemed to be coalescing around the role of social work researchers in furthering innovation as the core problem, with special attention to skills, competencies, and building relevant experience. This is a different problem from the goal of developing innovations or improving community health and well-being both of which extend beyond social work. Once the problems and goals are clear, mental models can begin to crystallize about how the problem has developed and how system experts believe it can be solved. Moreover, this step also clarifies the boundaries of the system in which the problem is occurring.

### Clarify the Boundaries of the System and Close the Loops

Clear boundaries of the system help to determine what is and is not within social work's power to address. For instance, scholars in all fields must contend with pressures to specialize and deepen knowledge. General academic pressures to do so are external influences on social work as a system, but if there are additional pressures, social work is unduly imposing on itself, that is an area worthy of discussion. This is especially the case because these pressures are seen as a key factor limiting the potential of social workers to become transdisciplinary researchers.

Related to the issues of boundaries are the many open loops in Figure 3. Open loops are identified by dead ends, as variables are followed around the loops. This leads to too few connections, for instance, between the loops on the lower left dealing with community and practitioner community and the loops on the upper right dealing with transdisciplinary partnerships. However, these partnerships have effects on each other, even if delayed or difficult to perceive; decisions made between and within these sectors is a primary source of complexity in the system.

### Identify Limits to Growth

As reflected in this article, there are many idealized loops depicting how the participants collectively imagined change positively occurring, a reflection of people's mental models. However, no system is governed completely by reinforcing loops; there are always limits to growth (Meadows, 2008). For instance, the reinforcing loop of community participation is slowed if researchers' reflexivity and ability to manage power imbalances is low or declines. These forces that push and pull are often acting simultaneously; relevant questions include “Which force is more dominant at any given time?” and “What can be done to shift the dominance?” These kinds of questions about what could hinder progress can be asked of many other loops in the system, once they are closed by clarifying the boundaries.

### Clarify Social Work's Unique Contributions

As noted early in the Results section, several participants mentioned the unique skills or potential contributions of social work, such as tools to manage power imbalances on teams; a strong, focused mission; and its ethos, holistic vision, and history of engagement with communities. These unique features of social workers have been noted elsewhere, as has the reluctance of social workers to use these skills in transdisciplinary settings (Gehlert, 2016). However, as with many other variables in Figure 3, the source of these skills was not always specified. It is not enough to rest on our laurels and assume that social workers are inherently more capable of addressing power, engaging in communities, or possessing holistic vision. If this is our unique contribution, then it should be explicitly named, defined, and be just as much a part of training as expanding expertise to new tools and technologies.

### Limitations

The analysis and recommendations are based on a high-level overview of innovation in social work; many important details were excluded to reach that level. The CLD was distilled through one author's analysis of concept papers written by 19 doctoral students and faculty members, all researchers, most of whom were trained in social work. Practitioners, educators, representatives from other fields and professions, and community members were not involved and would likely have different views. The original intent of these concept papers was not to develop a CLD. Had this been done in a group setting it likely would have resulted in a different diagram, because participants would have had the opportunity to debate and discuss the variables and links among them. The CLD is an initial step toward creating a boundary object or a visual representation of a problem that can be used to negotiate concepts and definitions among groups whose members may have different opinions about those concepts (Black & Andersen, 2012; Star & Griesemer, 1989). Whether the CLD succeeds as a boundary object—that is, it is relatable to readers—depends on at least two factors: the extent to which it is an accurate reflection of the group's thinking at the time of the innovation roundtable and the extent to which it is a representative reflection of the rest of the field of social work. Hopefully the text is relatable, even if the CLD is unfamiliar.

## Conclusion

Social work is not alone in its drive to develop innovative solutions to the world's complex problems. Transdisciplinary teams and ethical partnerships with communities and practitioners will be needed to develop innovative solutions that are of high quality and responsibly developed. Social work's historical mission of improving health and well-being through embeddedness in the community, its unique ethos and vision, and its ability to manage power should increase the confidence of a field that too often doubts itself. Continual development of these skills and unique attributes, when combined with new skills, is a key element necessary to develop 21st-century innovations. The field will need to determine how to capitalize on its existing talents while also creating the space, time, and resources to learn new skills and form new partnerships. This will require researchers, practitioners, and educators to make many decisions in a complex, ever-changing system. Principles and heuristics borrowed from system dynamics and other methods that account for complexity can improve decision-making as the field defines its own innovative science to tackle grand challenges.

## Figures and Tables

**Table 1:** Overarching Themes Derived From Review of Concept Papers and Construction of Causal Loop Diagrams.

Overarching Themes	Creating a Science of Social Work Through Research–Practice Partnerships	Grand Challenges, Responsible Innovation, and Social Work Science	Science, Innovation, and Social Work	Innovations in Community-Based and Interdisciplinary Research	Innovation in the Profession of Social Work Practice	Innovation and Emerging Scientific Careers
Outside factors changing academic expectations to be innovative	+	+	+		+	
Partnerships, collaborations, networks, and so on, as a key mechanism for innovation	+	+	+	+	+	+
Make-or-break factors in the success of transdisciplinary research		+		+		+
Social work's role in transdisciplinary partnerships		+	+	+		+
Make-or-break factors in the success of partnerships with practitioners and communities	+	+	+	+		+
Make-or-break factors in the success of innovative research	+	+	+	+	+	
Factors relevant to social work MSW and PhD training that could support or hinder success		+	+	+	+	+
